# Highly Mass-Sensitive Thin Film Plate Acoustic Resonators (FPAR)

**DOI:** 10.3390/s110706942

**Published:** 2011-07-04

**Authors:** Lilia Arapan, Gergana Alexieva, Ivan D. Avramov, Ekaterina Radeva, Vesseline Strashilov, Ilia Katardjiev, Ventsislav Yantchev

**Affiliations:** 1 Department of Solid State Electronics, Uppsala University, 751 21 Uppsala, Sweden; E-Mails: liar@angstrom.uu.se (L.A.); ilia@angstrom.uu.se (I.K.); 2 Department of Solid State Physics and Microelectronics, University of Sofia, 1164 Sofia, Bulgaria; E-Mails: gerry@phys.uni-sofia.bg (G.A.); ves@phys.uni-sofia.bg (V.S.); 3 Georgy Nadjakov Institute of Solid State Physics, 1784 Sofia, Bulgaria; E-Mail: eradeva@issp.bas.bg (E.R.)

**Keywords:** resonator, aluminum nitride, membrane, HMDSO, gravimetric, sensitivity

## Abstract

The mass sensitivity of thin aluminum nitride (AlN) film S0 Lamb wave resonators is theoretically and experimentally studied. Theoretical predictions based on modal and finite elements method analysis are experimentally verified. Here, two-port 888 MHz synchronous FPARs are micromachined and subsequently coated with hexamethyl-disiloxane(HMDSO)-plasma-polymerized thin films of various thicknesses. Systematic data on frequency shift and insertion loss *versus* film thickness are presented. FPARs demonstrate high mass-loading sensitivity as well as good tolerance towards the HMDSO viscous losses. Initial measurements in gas phase environment are further presented.

## 1. Introduction

A variety of physical, chemical and recently biochemical sensors based on the gravimetric principle utilizing electroacoustic devices have been around for years. When used, for example, as an organic gas sensor, the device surface is coated with a sensitive layer that reacts selectively to the sorption of gas molecules. As a result of the modified surface loading, the device changes its operation frequency. The principle of using acoustic wave mass sensitive transducers has initially been employed for thickness measurements of thin rigid films using the quartz crystal microbalance (QCM). QCMs have found wide application in chemical gas sensors due to their excellent frequency stability, low noise and high resolution [1,2]. During the past 20 years, micro-acoustic gas sensors utilizing various types of surface acoustic waves (SAW) have demonstrated improved performance [3]. As the wave field of SAW is confined to the vicinity of the substrate surface, their propagation characteristics become sensitive to any kind of surface perturbation and more specifically to mass loading. The detection is performed in either delay-line or resonator-stabilized oscillation, the latter being preferred in gas sensing due to the higher Q and lower device loss resulting in substantial noise reduction and resolution improvement. At present, these sensors are well known. Initial attempts to commercialize this technology have already been presented [4]. Quartz-based SAW gas sensors exploiting Rayleigh SAW (RSAW) and SH-SAW (Love, STW) have been extensively studied [5]. The results indicate sensor resolutions in the ppb range due to high sensitivity and very low noise levels [6,7]. Further, SH-SAW and QCM have also been extensively used in contact with liquid media [8] since shear waves are non-propagating (decaying) in liquids which in turn results in low wave emission losses and thus in retained device performance.

Contemporary thin film electroacoustic technology brought a new dimension to the acoustic wave sensors development. Thin film bulk acoustic resonators (FBAR) operating in the lower GHz range, employed in silicon-integrated miniaturized gas sensors, have demonstrated high mass sensitivity [9,10]. Acoustic plate modes (APM) represent another alternative to gravimetric sensing. Plate mode sensors have proved efficient in both gas and liquid [11–13] sensing.

**Figure 1.**
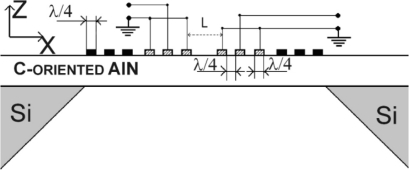
2 Port FPAR Schematic.

In this paper we study the mass sensitivity and the applicability of thin film plate acoustic resonators (FPAR) in gas sensing applications. FPARs represent a class of thin film acoustic devices utilizing the lowest order symmetric Lamb wave (S0) propagating in thin aluminum nitride membranes [14]. The S0 mode is known for its low dispersion, high velocity (10,000 m/s) and moderate electromechanical coupling [14] which make it a high velocity alternative to the conventional SAW. Furhter, the FPARs topology, while similar to that of the SAW-based devices (see Figure 1), is micro machined on AlN membranes on a silicon (Si) substrate [15]. Two-port FPARs have demonstrated insertion loss of around 3 dB and an unloaded Q of 3,000 at a frequency of 0.9 GHz [16]. Also, 0.9 GHz FPAR based oscillators have exhibited low phase noise [17]. These results, along with the expected high mass sensitivity of the S0 Lamb mode in acoustically thin plates [11,18], motivated us for the present study. Here the mass sensitivity of the FPAR is theoretically and experimentally studied in view of combining high sensitivity with low losses. The results are further supported by measurements in gas phase environment.

## 2. Theoretical Considerations

**Figure 2.**
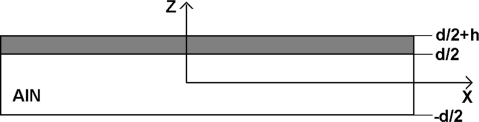
Geometry of the problem.

The mass sensitivity of the S0-Lamb wave in an acoustically thin plate loaded by a sensitive layer is described by using the equations of motion complemented by a full set of boundary conditions for a waveguided solution with polarization in the XZ-plane (see Figure 2). The S0 solution consists of two guided modes representing the shear and the longitudinal polarization, respectively, in each layer of the composite plate [19]. Subsequently, the equations of motion are applied to each layer of the composite plate along with the boundary conditions described below.

Normal stresses on the free plate surfaces (Z = −d/2 and Z = d/2 + h) are zero. Continuous particle displacements and normal stresses are required at the interface Z = d/2. These boundary conditions determine an eigen value—eigen vector problem for the eight unknown constants and the wave number β as a function of frequency. Alternatively, this set of equations can be solved for the acoustic wave velocity as a function of the wave number. Further details are omitted for brevity, since they have been thoroughly discussed elsewhere [19]. This approach has been verified using the results obtained by the Adler’s algorithm for lossless composite plates [20]. Mass sensitivity is assessed through the fractional change of the acoustic velocity as a function of the sensitive layer thickness. It is revealing to calculate and compare theoretically the mass sensitivity of the S0 Lamb wave to those of the RSAW and Love wave propagating on layer coated AT-cut quartz. The RSAW calculation can readily be done using the algorithm described in [21], while the Love wave calculation employs the layer boundary conditions derived in [22].

**Figure 3.**
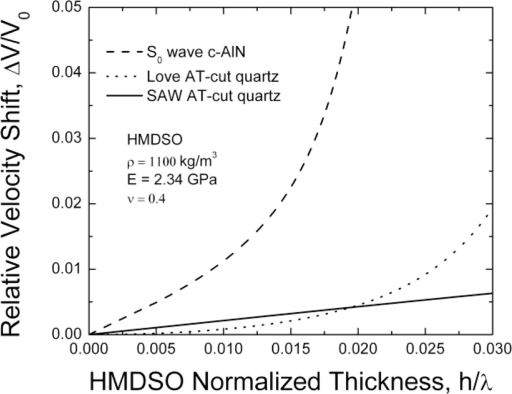
Theoretical mass sensitivity of RSAW, Love wave and S0 wave.

In this specific case an AlN acoustic plate with thickness-to-wavelength ratio d/λ = 0.167 as well as an AT-cut quartz substrate, both loaded with the glassy polymer pp-HMDSO are theoretically considered [23]. The calculated absolute fractional velocity shifts as a function of the pp-HMDSO relative thickness h/λ are shown on Figure 3. Evidently the S0 mode demonstrates a considerably higher mass sensitivity as compared to the RSAW and Love waves. However, increased sensitivity must not necessarily be the best choice for a practical system. Generally, improved mass sensitivity should correlate with improved damping especially at thicker layers [24]. Otherwise, the increased attenuation and decreased device Q could result in serious degradation of the oscillator noise and sensor resolution. This aspect is further theoretically studied for the S0 Lamb mode in comparison to RSAW and Love waves on AT-cut quartz. The above algorithms are applied presuming that the viscosity induced loss is represented by the ratio of the imaginary to real parts of the elastic constants (C”/C’). Here the coating layer was assumed to have effective elastic modulus C’ and viscosity η complying with Voigt’s visco-elasticity relation C= C’ + jC” = C’ + j2πfη. It is further to note that C”= 2πfη—scales with frequency and layer viscosity, while C’ is a frequency independent layer quantity.

**Figure 4.**
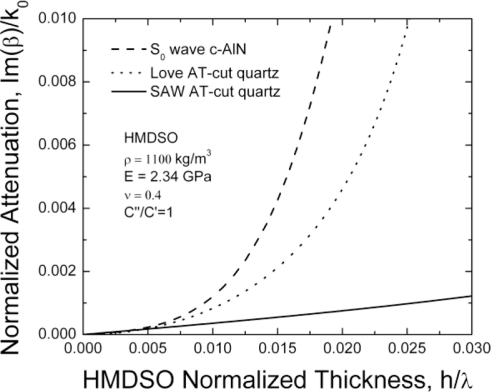
Attenuation of RSAW, Love and S0 waves. β-complex wave number, k0-real part.

In Figure 4 the attenuation of the studied modes is shown as a function of the pp-HMDSO layer thickness presuming C”/C’ = 1. The S0 mode attenuation is comparable to that of the RSAW and Love waves for relatively thin layers h/λ ≤ 1%. At larger layer thicknesses the S0 attenuation is significantly higher than the attenuation of the RSAW mode. The latter determines the frame where the use of S0 lamb wave can be beneficial. The use of relatively thin sensing films seems to be preferable, similarly to the case of surface transverse waves (STW) as compared to RSAW [24].

**Figure 5.**
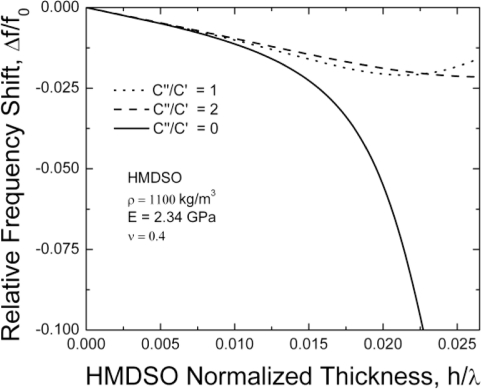
Theoretical mass sensitivity as function of layer viscosity.

**Figure 6.**
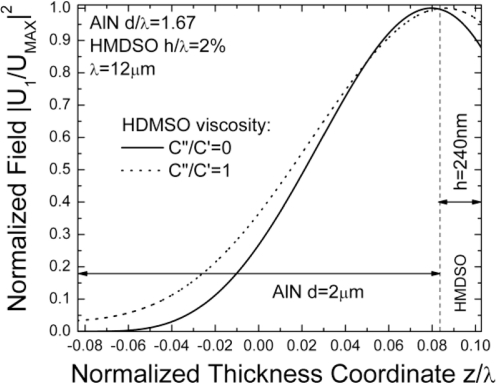
S0 Lamb wave confinement as function of the losses in the sensing layer.

Further, the layer viscosity itself influences the S0 mass sensitivity especially for pp-HMDSO layers with relative thicknesses h/λ > 1% (see Figure 5). In this range the sensitivity deteriorates significantly compared to the case of a lossless layer. Accordingly, the fractional frequency change *versus* layer thickness becomes lower and linear. This effect becomes clearer in Figure 6, where the field distribution within the composite plate of the dominant longitudinal component is shown as calculated by the algorithm proposed in this work. We see that the viscosity tends to decrease the energy trapping in the low impedance layer, which in turn reduces significantly the sensitivity at thicker layers. Quite a similar effect has been discussed for polymer coated SH-SAWs [21] although its nature has not been discussed so far. These results further confirm the conclusion that the S0-Lamb wave in acoustically thin plates could demonstrate high sensor sensitivities obtained with even relatively thin glassy sensitive layers.

## 3. FPAR Experimental Loading by pp-HMDSO

In this section we compare the theoretical relative frequency shifts of the S0-mode with experimental values. The experiments were performed with a 2-port FPAR with acoustic wavelength λ = 12 μm (grating pitch 6 μm). The FPAR device was micromachined on a freestanding AlN membrane with a thickness d = 2 μm resulting in a d/λ value of 0.1667. Then the FPAR has been coated with hexamethyldisiloxane (HMDSO)-plasma-polymerized thin films of various thicknesses. The actual thickness of the pp-HMDSO films has been measured during the deposition process by a QCM placed in close vicinity to the FPAR. The QCM has been calibrated by complementary thickness measurements with Talystep, Taylor-Hobson profilometer. After each deposition step the frequency shift as well as the insertion loss were recorded with a Vector Network Analyzer.

**Figure 7.**
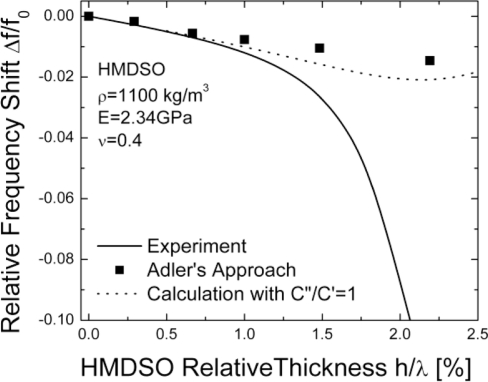
Theoretical *vs.* experimental mass sensitivity.

In Figure 7 the experimental frequency shift is compared to the theoretically determined shifts presuming lossless and lossy (C”/C’ = 1) pp-HMDSO films, respectively. For layer thicknesses h/λ < 1% the agreement between the lossless theory and experiment is excellent, while for thicker layers a progressive discrepancy is observed. The experimental data is seen to be in excellent agreement with the lossy theoretical predictions with C”/C’ ≈ 1. This point comes to support the loss of sensitivity mechanism discussed in the previous section (see Figure 5).

**Figure 8.**
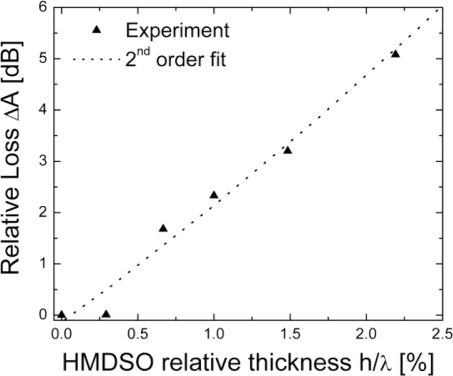
Change in insertion loss of a pp-HMDSO coated FPAR.

**Figure 9.**
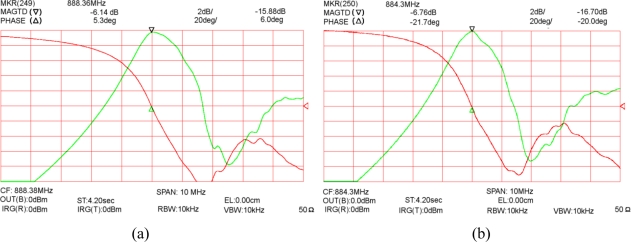
Narrowband frequency (upper curves) and phase (lower curves) characteristics of a 2-port FPAR measured (**a**) prior to layer deposition (**b**) after deposition of a 75 nm pp-HMDSO.

**Figure 10.**
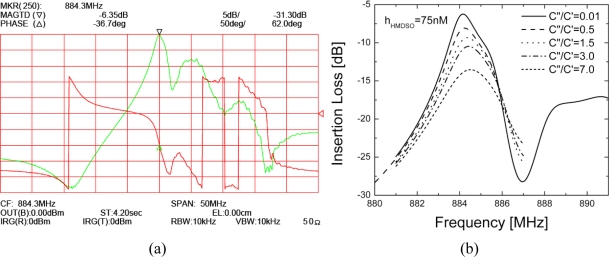
Frequency response of the 75 nm HMDSO coated FPAR (**a**) Wide frequency range measurements (**b**) FEM calculated close to resonance response.

In Figure 8 the recorded changes in FPAR insertion loss as a function of the pp-HMDSO thickness are shown. Relative losses of up to 2 dB are observed for layer thicknesses h/λ < 1%. Note that the close-to-resonance FPAR response is found mostly affected for layer thicknesses h/λ < 1% while the FPAR response away from the resonance looks unaffected by the layer losses. In Figure 9(a,b) the close-to-resonance frequency response of a 2-port synchronous FPAR is shown prior and after deposition of an acoustically thin pp-HMDSO layer with h/λ = 0.6%. The FPAR demonstrates around 4 MHz (4,600 ppm) frequency downshift at the expense of only 0.6 dB loss increase due to the HMDSO loading. The coated FPAR demonstrates an insertion loss of around 7 dB and a loaded Q of 700 (see Figure 10(a)). Note that an FPAR with such a performance was readily employed in a low noise frequency generator which is a prerequisite for achieving high measurement resolution. To further study the influence of the film losses on the FPAR response, finite elements method (FEM) analysis of the actual FPAR structure has been performed in a 2D approximation (device aperture effects neglected). COMSOL Multiphysics frequency response analysis of the complete 2-port FPAR structure consisting of 41 strips in each interdigital transducer and 52 strips in the reflectors has been performed. The waves emitted outside the FPAR structure have been absorbed without reflection using the perfectly matching layer feature available in COMSOL.

In Figure 10(b) close-to-resonance characteristics simulated for a 75 nM pp-HMDSO coated 2-port synchronous FPAR are presented. Note the excellent agreement between the FEM simulated and the experimental response. Again the frequency shifting effect of the layer viscosity is clearly seen although its influence on the device sensitivity is relatively weak. More importantly, this simulation suggests that FPAR devices coated with acoustically thin pp-HMDSO could sustain high viscous losses before significant performance degradation occurs.

## 4. Gas Sensitivity Experiments

**Figure 11.**
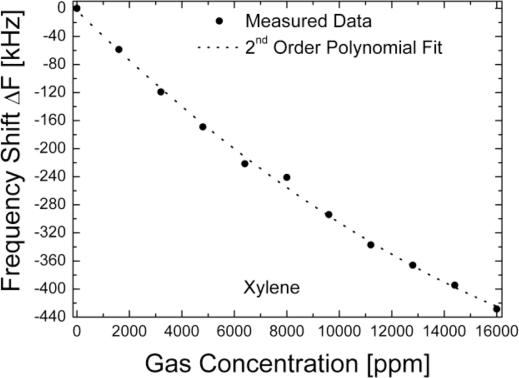
Sensitivity *vs.* xylene for the FPAR with 380 nm HDMSO.

The theoretical and experimental analysis performed in this work suggests that the FPAR is a highly sensitive alternative to RSAW and SH-SAW resonators. To strengthen this point we further considered a comparison of the FPAR gas sensitivity to that reported for other HMDSO-coated resonant devices using different types of acoustic modes. In [6] HMDSO-coated STW resonators at 700 MHz and SAW resonators at 430 MHz have been tested for their reaction against a number of gases including xylene. That study was aimed at achieving optimum HMDSO-layer thickness promoting maximum sensitivity. At the optimum thickness the wave energy is concentrated at the surface area of the film and strongly interacts with the sorbed gas [6]. Optimum values of HMDSO thickness were 100 nm (h/λ = 1.4%) for the STW case and 280 nm (h/λ = 3.9%) for SAW. The resulting sensitivities for xylene have been 6.4 Hz/ppm and 3.1 Hz/ppm, respectively. The conclusion drawn has been that STW-resonators have higher gas sensitivities at the same wavelength—a result that is in direct agreement with the theoretical comparison here (see Figure 3), although Love waves are chosen as a representative of the SH-SAW family. To check this comparison experimentally, an FPAR device with an HMDSO layer as thick as 380 nm (h/λ = 3%) was tested. The frequency downshift due to the layer deposition was 27 MHz (30,000 ppm). The HMDSO layer has not been optimized with respect to gas sensitivity and represents a relative thickness (h/λ) somewhere in between optimum values for STW and SAW, respectively. The device was gas probed with xylene vapors. The non-flow experimental method of applying the gas that we used for xylene probing has been described in detail in [25]. The changes of the resonant frequency and insertion loss have been recorded by a scalar network analyzer. After each step of adding a new amount of gas to the camera a time interval has been waited to expire to achieve equilibrium of the gas concentration and saturation of adsorption before registering the data. As the response of the resonators has been totally reversible, all data have been obtained on one and the same device. The experimental sensitivity of the 380 nm HMDSO coated FPAR device to xylene concentration is shown in Figure 11. The sensitivity curve is nearly linear with a 31 Hz/ppm slope which is about 5 times higher than the sensitivity of the STW resonator from [6], operating at about the same frequency. At the same time the FPAR loss varied within 1 dB over the whole concentration range which has a negligible effect on the sensor oscillator performance.

To relate the sensitivity values to the theoretical findings, the relative sensitivity to xylene defined as the ratio of the absolute sensitivity (in Hz/ppm) to the unloaded resonance frequency was calculated for FPAR, STW and SAW. These sensitivities were found to be in relation 5.0/1.3/1.0 for FPAR *vs*. STW *vs*. SAW, respectively, which is in good agreement with the theoretical expectations.

It is further noteworthy that HMDSO selectivity has also been assessed within this study. HMDSO coated FPARs have shown sensitivities towards ethanol, acetone and formalin in the range of about 1 Hz/ppm which is more than one order of magnitude lower than the sensitivity to xylene. The latter results seem reasonable if one bears in mind that HMDSO has been known as highly hydrophobic. Xylene molecules, also hydrophobic, should be adsorbed on the polymer surface at much larger quantities than the polar molecules of ethanol, acetone and formalin. Similar results have independently been observed in [26], where pp-HMDSO has demonstrated much higher sensitivity to the hydrophobic (non polar) toluene and diethyl ether as compared to the sensitivity to the polar acetone and ammonia.

The above explanation is further supported through gas probing experiments with acetic acid vapors. The HMSDO-coated FPAR has shown very high sensitivity of 120 Hz/ppm exhibited at low concentrations, up to 2,500 ppm followed by a linear part with slope of 25 Hz/ppm that extends to about 20,000 ppm gas concentration, where frequency reached the saturated downshift of −550 kHz. These features are also noticeable in the behavior of the loss whose increase again does not exceed the 1 dB range. A high reaction of HMDSO towards the acetic acid has been already established by QCM assisted experiments [26] but the effect has not been clarified. To look for a possible explanation, we repeated the check of the FPAR against xylene. It has turned out that: (i) the reference equilibrium frequency has been shifted downward by approximately 200 kHz; (ii) the overall gas sensitivity has decreased to 24 Hz/ppm. The most plausible reason for this kind of behavior is that the gas has produced a covalent bonding reaction on the polymer surface, thus increasing irreversibly its mass loading. As a second result of such a reaction, the hydrophobicity of the surface has been weakened leading to a decreased amount of adsorbed xylene molecules.

These initial results from the gas sensing experiments clearly support the theoretical predictions for high mass-sensitivity of the studied resonator type. The considerable sensitivity improvement over one of the most efficient acoustic wave mode gas sensors—the STW, for the same types of polymer film and probing gas, and at comparable frequencies and losses, reveals the great potential of the S0 mode for sensor applications. Another issue of practical importance is the fact that this high sensitivity is accompanied by a weak effect on the resonator loss even at high concentrations of aggressive gas phase compounds interacting with the polymer coated surface. The authors believe that these features may be attractive for integrated circuit compatible FPAR sensor systems in the near future.

Further research is needed to explore the effects of membrane scaling on the sensor performance. Note that the weak dispersion of the S0 Lamb mode [14] allows one to improve the sensitivity of the FPAR without any change of the resonant frequency, *i.e*., decreasing the membrane thickness. This unique opportunity has also been discussed in contour-mode resonant configurations employing the same mode [27]. More generally, the improvement in sensitivity is expected to be combined with reduced affinity to the viscous losses in the sensitive layer which in turn imposes stronger limitations on the gas sensitive coating thickness. The latter applies also to frequency scaling where the used membranes are thinner and the layer losses larger. In other words, the optimum FPAR sensor design is application specific, *i.e*., requires a trade off between FPAR mass sensitivity and layer losses in order to achieve maximum resolution.

## 5. Conclusions

The mass sensitivity of thin film plate acoustic resonators has been experimentally and theoretically studied. The influence of the sensing layer visco-elasticity over the device performance has been specifically addressed. Generally, the thin film plate acoustic resonators appear to exhibit specific advantages with respect to sensitivity and resolution under the condition that acoustically thin visco-elastic sensing layers are used. It has been proved that the symmetric Lamb mode used is considerably better in sensitivity compared to other acoustic modes such as SH-SAW or RSAW. As a result, in a gas sensing high frequency configuration the experimental sensitivity to a specific analyte has reached up to five times that obtained with other sensitive acoustic devices. The results highlight the potential of the FPAR in gas sensing applications in view of designing on chip high resolution sensor arrays for multiple gas detection.
